# Impact of Increased Respiratory Load on Prefrontal Cortical Activation Under Varying Task Complexities of Trail Walking Task

**DOI:** 10.3390/s26185834

**Published:** 2026-09-15

**Authors:** Alka Bishnoi, Manuel E. Hernandez

**Affiliations:** 1 School of Kinesiology, Auburn University, Auburn, AL 36849, USA; 2 Carle Illinois College of Medicine, University of Illinois at Urbana-Champaign, Urbana, IL 61801, USA; mhernand@illinois.edu

**Keywords:** breathing rate, aging, fNIRS, dual tasking, prefrontal cortex

## Abstract

This study examined the effect of breathing rate (BR) on prefrontal cortical (PFC) activation during single-task and dual-task (e.g., Trail Walking Task (TWT)) conditions in a diverse aging population. We hypothesized that higher BR would be associated with increased PFC activation, particularly during dual-task walking. In this cross-sectional study, 45 adults (mean age 50.31 ± 20.19 years; 27 females) walked on an instrumented treadmill at a self-selected pace under single-task and TWT conditions. Resting BR was measured using a smart shirt, and functional near-infrared spectroscopy assessed PFC oxygenated hemoglobin (HbO_2_) and deoxygenated hemoglobin (Hb) during TWTA (numbers only) and TWTB (numbers and letters). Linear mixed-effects models evaluated the effects of BR, task, and their interaction on PFC activation, controlling for age and cardiorespiratory fitness (VO_2_ max). The results showed significant differences in Hb and HbO_2_ between single- and dual-task conditions across BR levels. Individuals with higher BR exhibited greater PFC activation during TWT, whereas lower BR was associated with reduced activation and more efficient attentional resource allocation. BR was not associated with walking speed. These findings suggest that higher BR reflects increased neural effort during dual-task walking and highlight breathing as a potential modifiable factor influencing cognitive-motor performance and fall risk.

## 1. Introduction

Breathing is a fundamental physiological process that extends far beyond gas exchange, playing a critical role in neural regulation, autonomic control, and motor coordination. Respiratory rhythms are tightly coupled with brain activity, influencing neural oscillations, cognitive performance, and movement execution [1,2]. Diaphragmatic breathing has been shown to reduce stress, improve cardiovascular efficiency, and enhance respiratory mechanics, with emerging evidence suggesting its relevance for optimizing both cognitive and motor performance during locomotion [2,3,4]. These effects are especially salient during dual-task walking, where cognitive demands interact with gait control to challenge system-wide physiological reserves [5].

Functional near-infrared spectroscopy (fNIRS) research consistently demonstrates that walking under increasing cognitive or physical load is associated with elevated prefrontal cortex (PFC) activation [6,7]. While this heightened activation reflects compensatory neural recruitment to preserve gait stability, it also signifies an increased reliance on executive resources. Such overrecruitment is particularly evident in older adults and individuals with neurological impairments, including stroke survivors, and may represent inefficient neural processing that ultimately constrains functional mobility [8,9]. Breathing load introduces an additional, yet underexplored, dimension to this cognitive-motor interplay.

Respiration has been shown to entrain cortical neural oscillations, synchronizing sensory, cognitive, and motor processes with the breathing cycle [1]. Respiration-locked oscillatory activity, particularly in the gamma band, may optimize neural efficiency during complex motor tasks and under conditions of increased load [1]. However, inefficient respiratory patterns or excessive breathing effort can disrupt this synchronization, increasing cognitive strain and potentially destabilizing gait [10]. Collectively, these findings underscore the importance of respiratory load as a modulator of prefrontal activation, gait performance, and energy expenditure across populations, with implications for fall risk, functional decline, and rehabilitation strategies.

Breathing is therefore not merely a metabolic necessity but a bidirectional marker of cognitive load and physiological arousal [11,12,13]. During walking, this interaction is mediated by locomotor–respiratory coupling (LRC), whereby synchronization between stride and breath minimizes the work of breathing [14,15]. Age-related declines in pulmonary compliance and gas exchange efficiency can disrupt this coupling [15]. Moreover, deviations from preferred stride frequency, such as those imposed by dual-task walking, significantly increase oxygen consumption [16], potentially creating a metabolic competition between respiratory musculature and prefrontal cortical processing. Stress-induced hyperventilation may further exacerbate this effect by inducing hypocapnia-related cerebral vasoconstriction, independently altering fNIRS-derived HbO_2_ signals [17,18,19].

Given the gap in our understanding of the relationship between pulmonary and cerebrovascular function as we age, this study aimed to evaluate the relationship between breathing rate and prefrontal cortex activation during dual-task walking. Through the integration of neuroimaging, motor performance, and cognitive measures within a novel dual-task framework, this study offers insight into pulmonary and cerebrovascular functional changes in cognitively demanding conditions in adults across the lifespan. Building on emerging evidence linking autonomic reserve to cortical activation [20], we hypothesize that increased breathing rate will be significantly associated with elevated PFC activation during cognitively demanding walking tasks. Such a relationship would suggest that respiratory effort could serve as a measurable peripheral marker to examine neural cost required to manage cognitive-motor interference, offering a novel physiological lens through which mobility resilience and early functional decline may be understood.

## 2. Methods

### 2.1. Participants

**Figure 1:**
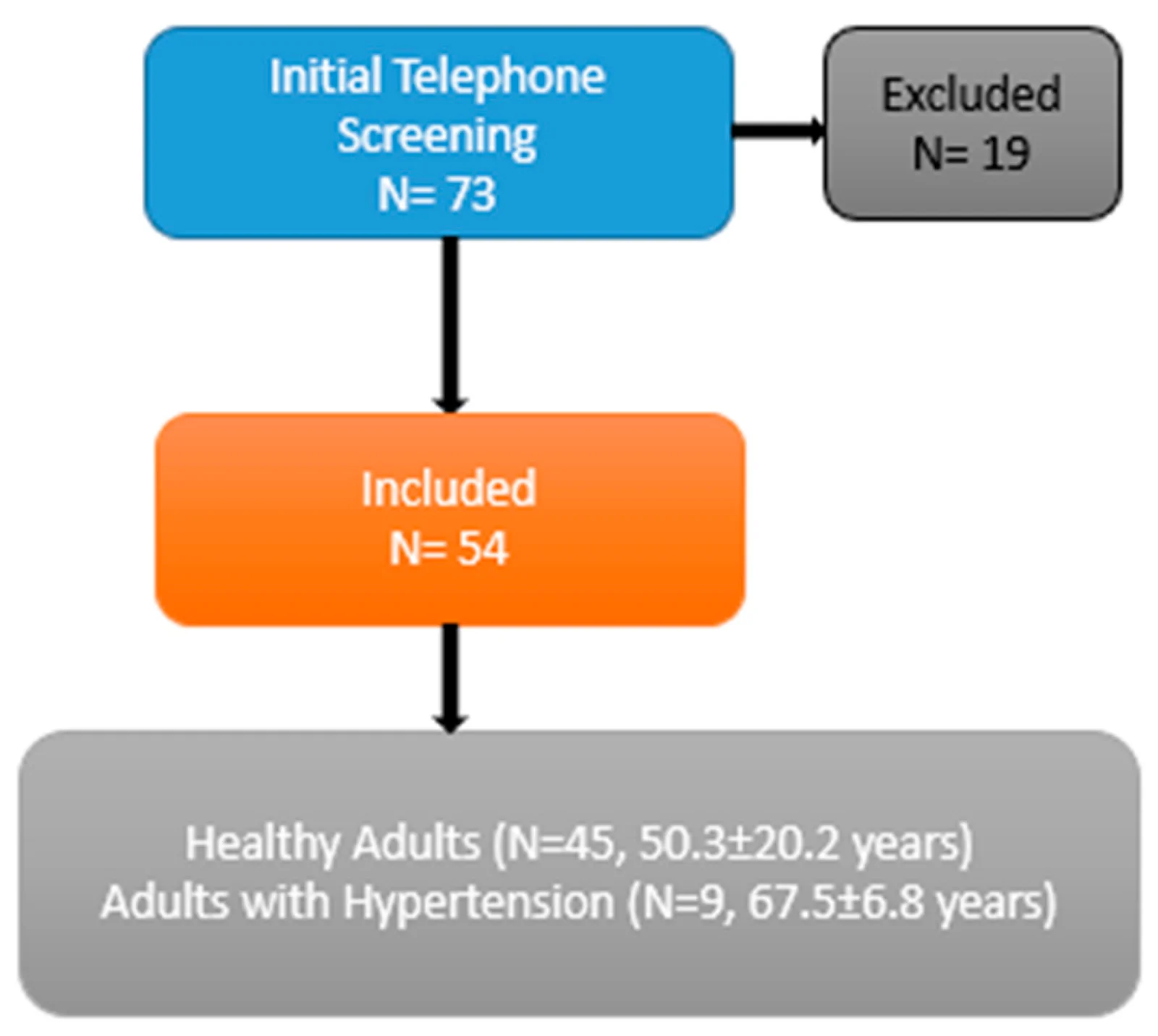
Participant recruitment and study flow diagram.

A total of 45 adults participated in this study (mean age: 50.31 ± 20.19 years), including 27 females. Participants were eligible if they were between 18 and 75 years old and reported no history of clinically diagnosed neurological, orthopedic, or cardiovascular conditions. Individuals were excluded if they demonstrated cognitive impairment, defined as a Telephone Interview for Cognitive Status score below 22 [21]; reported any neurologic, orthopedic, or cardiovascular condition; had physical disabilities; were unable to ambulate independently (e.g., required an assistive device); or had been hospitalized within the previous six months. Prior to initiating the study procedures, all participants reviewed and signed informed consent (Figure 1). The research protocol adhered to the principles outlined in the Declaration of Helsinki and received approval from the institutional review board at the study site.

### 2.2. Protocol

**Figure 2:**
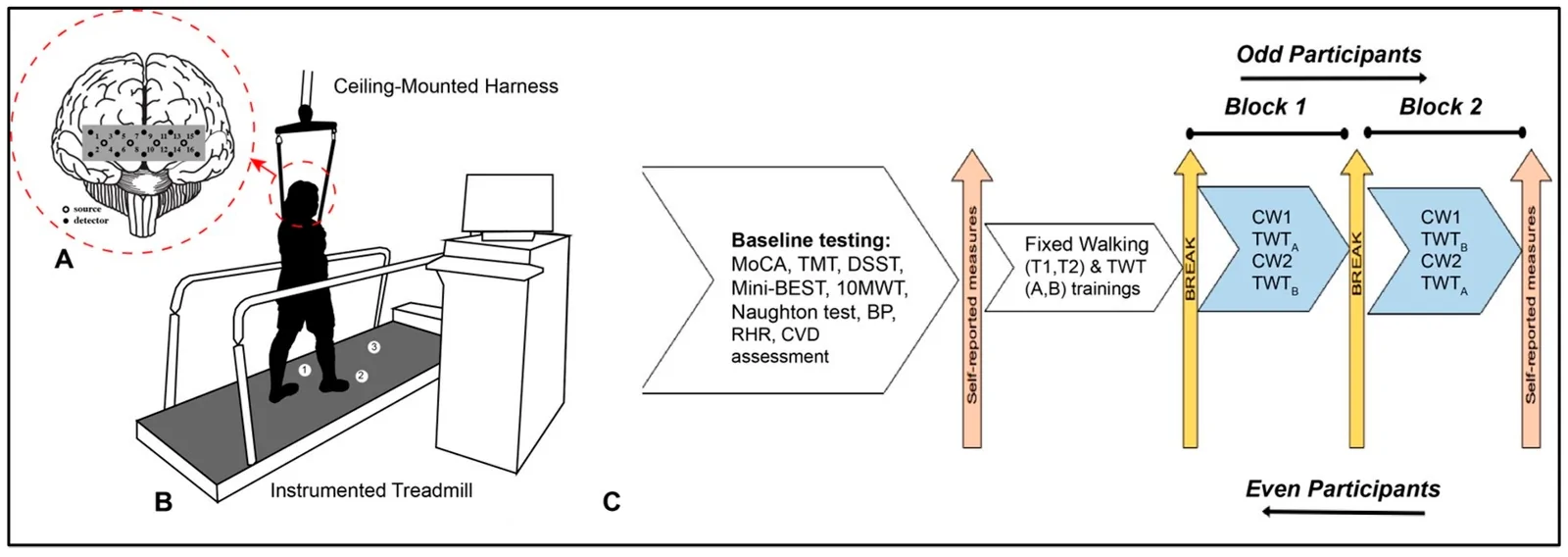
(**A**) Placement of fNIRS sensors over the prefrontal cortex; (**B**) experimental setup illustrating the Trail Walking Task (TWT); (**C**) summary of the study protocol. Abbreviations: CW = comfortable walking; TWTA = Trail Walking Task A; TWTB = Trail Walking Task B; MoCA = Montreal Cognitive Assessment; Mini-BEST = Mini-Balance Evaluation Systems Test; RHR = resting heart rate.

Participants attended a single laboratory session for data collection (Figure 2). During the visit, they completed baseline assessments that included cognitive and physical performance measures such as the Montreal Cognitive Assessment (MoCA) [22], Mini-Balance Evaluation Systems Test (Mini-BEST) [23], and Naughton submaximal exercise test [24], along with cardiovascular measures including resting heart rate and age-predicted maximum heart rate. Following baseline evaluation, the researcher established each participant’s comfortable walking speed by initiating treadmill movement at 0.7 m/s and gradually increasing the pace until the participant reported it felt appropriate, neither too fast nor too slow. Once this comfortable walking (CW) speed was determined, participants completed a five-minute acclimatization session at this pace on an instrumented treadmill (C-Mill, Motekforce link, Culemborg, The Netherlands), followed by two brief practice sessions of the Trail Walking Tasks A and B (TWTA and TWTB) prior to the experimental trials involving fNIRS. Subsequently, participants performed two experimental blocks consisting of single-task walking (walking at fixed speed for two minutes on the treadmill) and dual-task conditions (TWTA and TWTB) while outfitted with an fNIRS headband and a Hexoskin smart shirt (Carre Technologies Inc., Montreal, QC, Canada).

Both experimental blocks were completed on the same day, with a rest interval incorporated to minimize physical and cognitive fatigue. The sequence of dual task conditions (TWTA and TWTB) was counterbalanced across blocks to reduce potential order effects. Physiological responses, including breathing rate (BR) and heart rate (HR), were recorded during CW, TWTA, and TWTB across both blocks using the Hexoskin smart shirt, a device with demonstrated reliability and validity for measurements [25]. At the conclusion of the session, participants completed self-reported questionnaires assessing balance confidence, fall history, depressive symptoms, educational background, and activities of daily living.

### 2.3. Dual-Task Walking Paradigm

The experimental design included two conditions, namely, (1) single-task walking and (2) dual-task walking, represented by Trail Walking Tasks (TWTs). During dual-task conditions, the TWTs were administered in a graded sequence of difficulty, beginning with numbers only (TWTA) and progressing to a combination of numbers and letters (TWTB) in the first block, while the order was reversed in the second block. All walking tasks were completed using the C-Mill instrumented treadmill. This treadmill system allowed for continuous, real-time adjustments in speed, ensuring a safe, adaptive, and comfortable walking experience for participants.

Throughout all walking trials, participants were secured with a safety harness and instructed to maintain their predetermined comfortable walking (CW) speed established during the initial training session. Each walking trial started with a brief period to allow participants to achieve their CW speed before data collection began. For dual-task trials, tasks were initiated only after participants reached their steady CW pace (Figure 2).

### 2.4. Instrumented Trail Walking Task (TWT)

During the TWT, augmented reality visual cues and distractors were projected onto the treadmill surface (3 m × 0.7 m), with presentation rates tailored to match each participant’s comfortable walking speed [20]. The TWT protocol included two distinct conditions administered across two blocks: Trail Walking Task A (TWTA) and Trail Walking Task B (TWTB). Tasks within each block were pseudorandomized to reduce potential learning or practice effects. Each block comprised the following conditions: two minutes of fixed comfortable walking (CW), TWTA, and TWTB, with all tasks performed in both blocks. TWTA involved stepping on numbered targets ranging from 1 to 30, interspersed with distractor stimuli. Participants were instructed to step on the numbers in sequential ascending order while avoiding distractors. Task accuracy was evaluated for targets between numbers 5 and 30. Cognitive performance was assessed by trained research assistants and subsequently verified through video recordings collected during the session. Separate counts were maintained for missed targets and incorrect steps on distractors for each task. TWTB introduced a higher level of complexity by incorporating both numbers and letters as targets, with similar distractors presented between them. Participants were instructed to step on alternating numbers and letters in ascending order, like the Trail Walking Test, while avoiding distractors. Accuracy for TWTB was assessed between the sequences 5–E and 26–Z.

The order of blocks (Block 1 and Block 2) was reversed for even-numbered participants to control for sequencing effects. Additionally, tasks differed slightly between blocks, and their order was alternated across blocks to further reduce order-related bias. In Block 1, tasks were performed in the sequence CW → TWTA → CW → TWTB, whereas in Block 2, the order was CW → TWTB → CW → TWTA. Even-numbered participants completed these blocks in reverse order, beginning with Block 2 followed by Block 1. Overall, the use of counterbalancing and pseudorandomization strategies was implemented to minimize learning and order effects.

### 2.5. fNIRS Data Acquisition

fNIRS signals were acquired using the fNIRS Imager 1200 system (fNIRS Devices, LLC, Potomac, MD, USA). The headband incorporated 4 LED light sources and 10 photodetectors arranged across the forehead, forming 16 optode channels with a source–detector separation of 2.5 cm and a sampling frequency of 2 Hz. The light emitters (Epitex Inc. (Singapore), model L6 × 730/6 × 850) consisted of dual-wavelength LEDs with a 730 nm and 850 nm peak wavelength and an external diameter of 9.2 ± 0.2 mm. The photodetectors (Burr Brown, model OPT101) functioned as monolithic photodiodes integrated with a transimpedance amplifier powered by a single supply. The headband was positioned such that its midpoint aligned with the Fpz location, corresponding to the center of the forehead above the nasion according to the international 10/20 EEG system. Relative concentrations of oxygenated (HbO2) and deoxygenated hemoglobin (Hb) (μM) were analyzed, given their established reliability in reflecting cortical activation changes [26].

**Figure 3:**
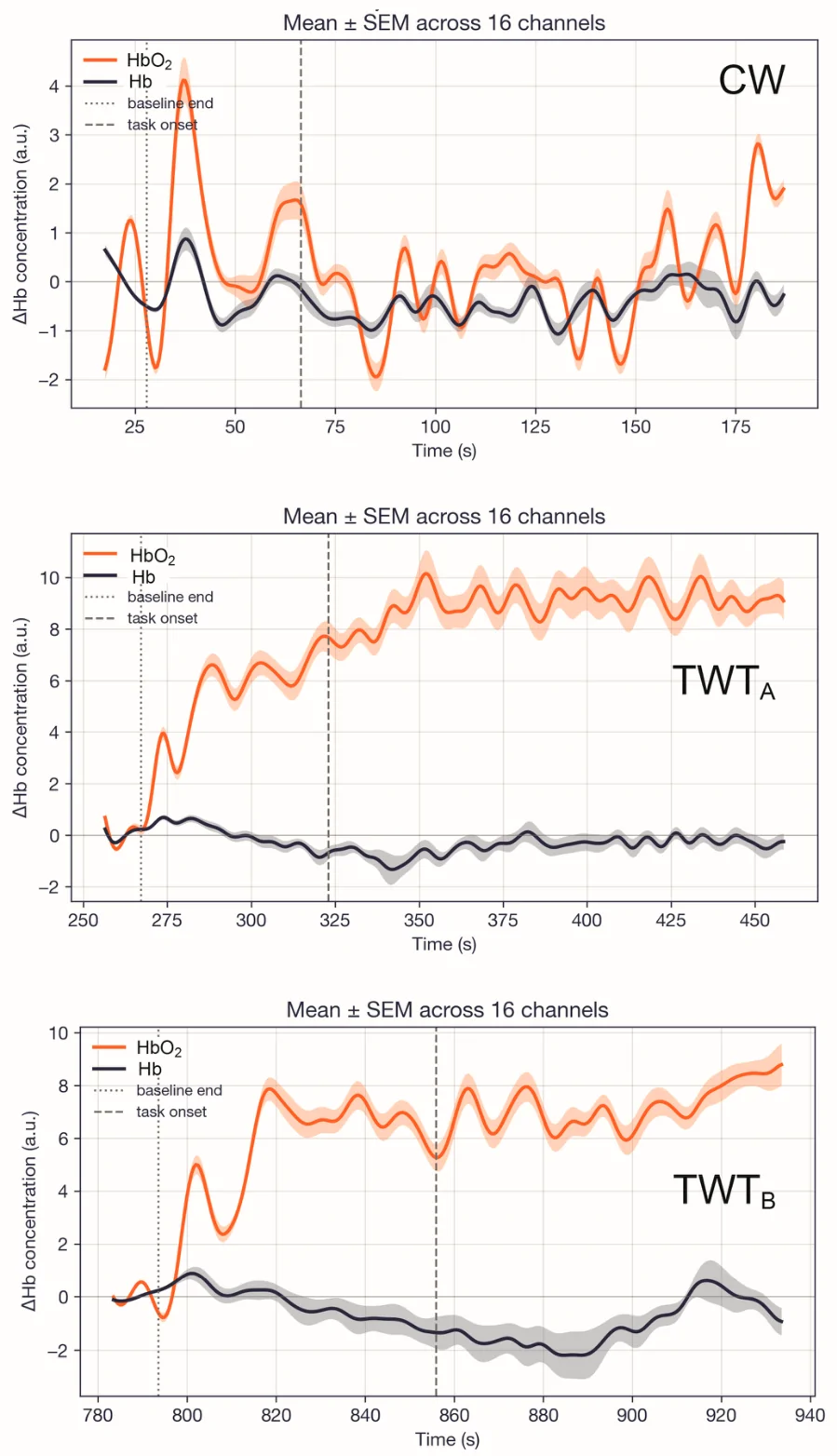
Representative participant-level fNIRS time-series signals across experimental tasks. Mean (SEM) oxyhemoglobin (HbO_2_) and deoxyhemoglobin (Hb) levels recorded from all 16 fNIRS channels from baseline to end of (**top**) comfortable walking (CW), (**middle**) Trail Walking Task A (TWT_A_), and (**bottom**) Trail Walking Task B (TWT_B_).

Data acquisition was performed using COBI Studio software (v1.3.0.19), and subsequent processing and analysis were carried out with custom MATLAB scripts. Raw signal quality was initially evaluated through visual inspection to identify potential issues such as noise, signal saturation, or dark current artifacts. To reduce the influence of physiological (e.g., respiration and cardiac activity) and other sources of noise, a low-pass filter with a cutoff frequency of 0.14 Hz was applied to the data [27]. Concentrations of HbO_2_ and Hb (μM) were then derived for each of the 16 channels using the modified Beer–Lambert law [28] (Figure 3).

Prefrontal cortex (PFC) activation was evaluated across both experimental blocks during the conditions of comfortable walking (CW), TWTA, and TWTB. Task duration varied among participants because walking pace was individualized based on their predetermined comfortable speed while completing a fixed course for TWTA and TWTB. Mean PFC activation was calculated using the entire duration of each task. Each trial included a 10 s standing baseline period at both the beginning and end of the task. During this baseline interval, participants were instructed to look straight ahead and silently count starting from one. Following this baseline period, task-specific instructions were provided. The pre-task 10 s baseline was used as the reference condition for calculating relative changes in HbO_2_ and Hb concentrations (μM) [29].

### 2.6. Statistical Analysis

Custom MATLAB scripts were used to export the dataset into R (version 3.1.1). Spatiotemporal outputs generated in CueFors 2 (Motekforce Link, Culemborg, The Netherlands) were transferred to Python for further handling. We then processed the spatiotemporal variables using custom Python code and exported the cleaned outputs into RStudio (version 3.1.1). The alpha level for statistical significance was defined as 0.05. Age and aerobic fitness were included as a covariate based on their use in previous fNIRS-related analysis [30]. To test our hypothesis, we modeled breathing rate (BR) as a continuous independent measure using a linear mixed-effects framework, with task condition (CW vs. TWTs) as a categorical independent measure to evaluate differences in PFC activation, our dependent measure, while controlling for repeated measures, across all 16 channels, and each subject modeled as a random factor, with their own random intercept. Data were screened visually, and the linear mixed-model assumptions were satisfied. When assumption violations occur for any outcome, we applied a rankit transformation to normalize the distribution [31]. In this analysis, rankit transformation was specifically applied to mean oxygenated and deoxygenated hemoglobin values. To adjust for multiple comparisons, post hoc pairwise tests were conducted in R using lsmeans with Tukey’s adjustment. All mixed-effects models were fitted in R (version 3.1.1; R Development Core Team, 2014) using the lme4 package [32].

## 3. Results

### 3.1. Participants

A total of 45 adults who did not report any diagnosed medical conditions participated in this study. Participant demographics and baseline characteristics are summarized in Table 1.

### 3.2. Prefrontal Cortical Activation

**Figure 4:**
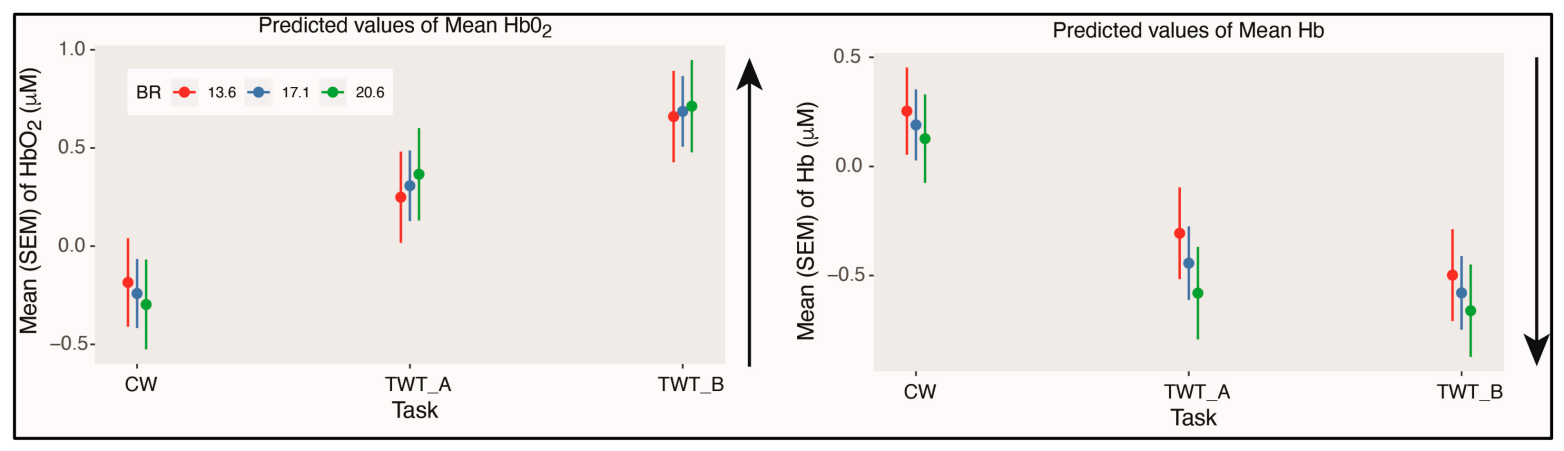
Mean ± SE of PFC activation for each condition (CW, TWT_A_, TWT_B_). The arrow denotes the direction of increasing PFC activation for mean HbO_2_ and Hb levels. Abbreviations: CW = comfortable walking, TWT_A_ = Trail Walking Task A, TWT_B_ = Trail Walking Task B.

**Figure 5:**
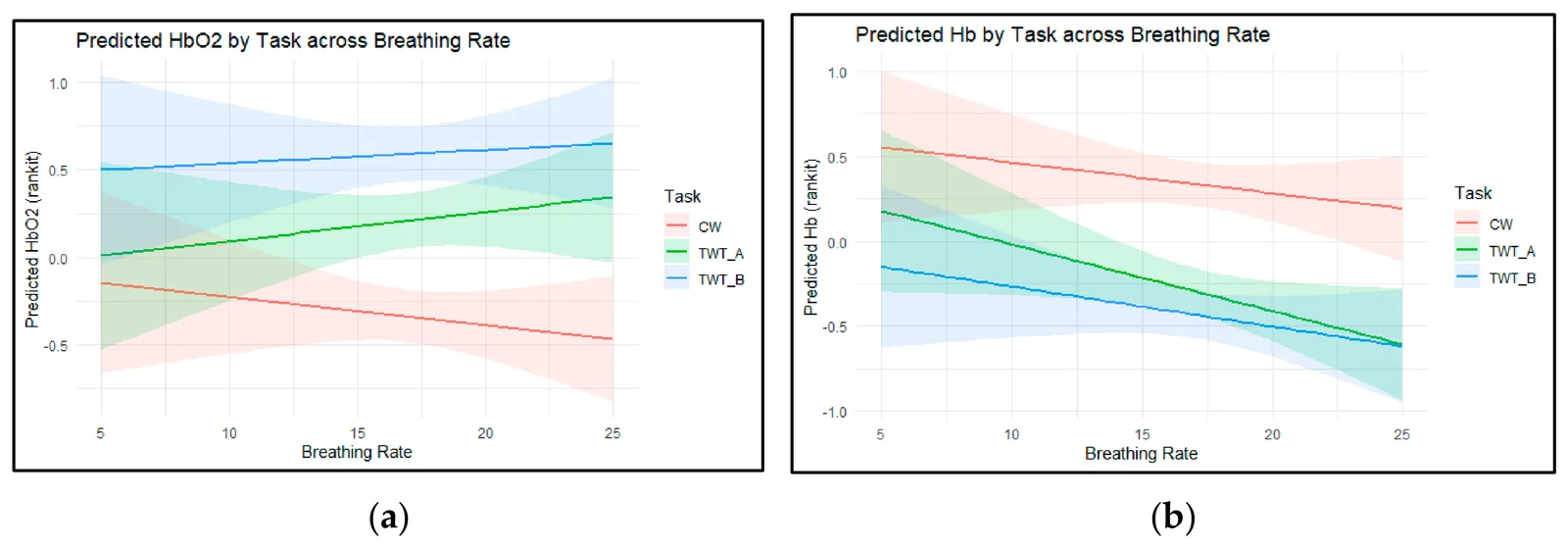
Model-predicted (**a**) HbO_2_ and (**b**) Hb concentration by task condition across the observed range of breathing rate (breaths/minute).

Prefrontal cortex activation (Hb [μM] and HbO_2_ [μM]) during single- and dual-task walking, averaged across both blocks, is shown in Figure 4. Linear mixed-effects modeling revealed significant main effects of task (*p* < 0.01) and significant breathing rate (BR) × task interaction effects (*p* < 0.05) for mean Hb and mean HbO_2_ (μM) (Table 2, Figure 5), after adjusting for age and fitness (VO_2_max). In general, a higher BR was associated with larger PFC activation, reflected by greater mean HbO_2_ and Hb during the more demanding TWT conditions compared with CW. Post hoc testing further delineated these interaction patterns. Relative to CW, both TWT_A_ and TWT_B_ elicited elevated PFC activation, with an additional increase observed from TWT_A_ to TWT_B_ (*p* < 0.0001).

## 4. Discussion

This study enhances our understanding of the relationship between breathing rate (BR) and executive function by integrating neuroimaging techniques with dual-task paradigms in a diverse aging population. Extending findings from earlier research demonstrating the impact of respiratory load on both cognitive and motor processes under different conditions [1], the present study specifically explored how BR is associated with neural activity during dual-task walking. Coordinating breathing with movement to maintain mobility is now considered a cognitively demanding activity, rather than a process governed solely by automatic motor control [1].

Age-related declines in executive capacity further impair the ability to simultaneously perform cognitive and motor tasks, a phenomenon known as cognitive-motor interference (CMI) [33,34]. This interference is particularly evident during the Trail Walking Test (TWT) in our study, which requires individuals to navigate a walking path while performing continuous visual scanning, sequencing, and mental flexibility tasks [20,35]. Performance decrements during the dual task often preceded overt cognitive decline and reflect disrupted postural control following cerebral insult [36,37]; identifying early physiological markers of CMI in middle-aged adults is critical for preventative intervention. To maintain stability during high-demand tasks such as the TWT, the aging brain frequently engages compensatory neural mechanisms. The Compensation-Related Utilization of Neural Circuits Hypothesis (CRUNCH) posits that older adults increasingly recruit PFC resources to meet task demands that younger adults can manage more efficiently [38,39].

Importantly, PFC recruitment follows a non-linear trajectory; beyond a certain threshold of task difficulty, cortical activation reaches a ceiling, after which performance deteriorates. Such patterns have been observed across healthy aging and neurological conditions including Parkinson’s disease and stroke [40,41]. Despite fNIRS providing a direct window into cortical load [7], the concurrent peripheral physiological costs, particularly respiratory and autonomic demands, remain poorly characterized. This gap may be addressed through the Neurovisceral Integration Model, which posits that the PFC exerts top-down regulation of autonomic function, including respiratory and cardiac rhythms, via vagal pathways [42,43].

### 4.1. Primary Findings and Theoretical Interpretation of PFC Activation Differences

The present study investigated how breathing rate (BR) influences prefrontal cortex (PFC) activation, measured using HbO_2_ and Hb signals, during dual-task walking in a heterogeneous age group using an instrumented Trail Walking Task (TWT) paradigm. In line with our hypotheses and previous research [1,20,30], dual-task conditions resulted in significantly higher HbO_2_ levels and reduced Hb levels compared with single-task walking. These findings align with prior research on autonomic engagement, which has shown greater recruitment and activation of the PFC during dual-tasking relative to single-task locomotion [20]. Participants exhibiting higher BR demonstrated increased neural activation and a pattern indicative of motor-cognitive facilitation, in contrast to those with lower BR, who showed patterns consistent with interference, even after adjusting for age and fitness levels.

These results support the Compensation-Related Utilization of Neural Circuits Hypothesis (CRUNCH), which proposes that aging is associated with increased neural resource recruitment to meet task demands that younger individuals can perform with fewer resources [38,39]. In contrast, individuals with lower BR appeared unable to recruit sufficient neural resources to sustain task performance, suggesting limited compensatory capacity. Across all participants, PFC activation increased as both motor and cognitive demands intensified (e.g., transitioning from standing to walking and to more complex dual tasks), which is consistent with earlier findings in younger [5] and older adults [44]. Additionally, similar to prior work reporting greater PFC activation in older versus younger individuals [29], our results showed that advancing age was associated with higher PFC activation across all walking conditions.

The consistently elevated PFC activation observed in participants with higher BR across task conditions may be partially explained by evidence indicating that greater fitness levels are linked to improved cerebral oxygenation and perfusion, whereas lower fitness is associated with reduced oxygen delivery and less efficient cerebral blood flow [45,46,47]. For this reason, both age and fitness were included as covariates in the analysis; notably, the association between BR and PFC activation remained significant after accounting for these factors. Given the well-established relationship between cardiopulmonary function and cerebrovascular health, the reduced and more uniform PFC activation patterns observed in individuals with lower BR may reflect comparatively diminished cerebrovascular efficiency. This reduced activation pattern may also be interpreted within the framework of neural compensation, where alternative neural networks are recruited to offset deficits in primary networks [48], although further investigation using broader neuroimaging coverage is required to confirm this explanation.

### 4.2. Limitations

Several limitations should be considered when interpreting these findings. First, the relatively small sample size, likely influenced by data collection constraints during the pandemic, may limit generalizability, despite attempts to match participants on age, sex, and physical function. Future studies should recruit larger, well-matched cohorts to strengthen external validity. Second, because participants did not exhibit cognitive impairment, the results cannot be readily extended to populations with cognitive deficits. Subsequent research should include more diverse groups, such as individuals with cognitive impairment or cardiovascular disease, and incorporate advanced biomarkers or neuroimaging techniques to better understand underlying mechanisms linking cardiovascular function, brain activation, and mobility.

Another limitation is that fNIRS measurements were confined to the PFC, restricting insight into other cortical and subcortical regions that may contribute to dual-task performance; future work should include broader neuroimaging coverage to further evaluate neural compensation mechanisms. Through the use of a larger number of sensors to improve spatial coverage, fNIRS and electroencephalography could be used to monitor other cortical areas involved in dual-task performance, such as the occipital, parietal, and motor cortices [49,50]. Further, through the combination of higher-dimensional neuroimaging data, motor and cognitive outcome measures, and machine learning approaches, further insights into early changes in respiratory and cerebrovascular function may be identified [51]. Additionally, although this study identified associations between BR and PFC activation during dual-task conditions, the cross-sectional design precludes any causal conclusions. Longitudinal or intervention-based studies are needed to determine whether modifying BR can enhance cognitive-motor integration or mitigate age-related decline. Accordingly, future research should explore intervention strategies aimed at improving BR to examine their impact on cognitive-motor interference.

## 5. Conclusions

In conclusion, this study evaluated the relationship between breathing rate and prefrontal cortex activation during dual-task walking. By integrating neuroimaging, motor performance, and cognitive measures within a novel dual-task framework, these findings offer insight into early changes in pulmonary and cerebrovascular function that may be modifiable through targeted interventions. This work underscores the value of combining neuroimaging with cardiopulmonary assessments to identify early dysfunction that could influence both quality of life and long-term health outcomes.

## Figures and Tables

**Table 1:** Demographic and characteristics of participants.

Characteristics	N = 45, 27 Females
Age (years)	50.31 ± 20.19
BMI (kg/m^2^)	23.35 ± 3.96
MoCA (max 30)	27.4 ± 1.98
Mini-BEST Score (max 28)	24.93 ± 1.49
Walking Speed (meter/s)	1.14 ± 0.19
Breathing Rate (breaths/min)	17.05 ± 3.62

Note: Mean ± standard deviation (SD) is shown. N, number of participants; BMI, body mass index; MoCA, Montreal Cognitive Assessment; Mini-BEST, Mini-Balance Evaluation Systems Test.

**Table 2:** BR and task effects on HbO_2_ (μM) and Hb (μM) levels after controlling covariates (age and fitness levels).

Effects	Estimate	SE	t-Value	95%CI Lower	95% CI Upper	df	*p*-Value
Mean HbO_2_ (μM)							
Intercept	1.168	0.54	2.169	0.084	2.248	40.15	0.036 *
Age	0.0035	0.003	0.92	−0.004	0.011	38.18	0.36
BR	−0.016	0.02	−0.763	−0.058	0.026	40.67	0.45
Vo2 max	−1.046	0.26	−3.91	−1.58	−0.51	38.88	0.0003 ***
Task: TWTA	−0.011	0.16	−0.068	−0.33	0.31	3321	0.945
Task: TWTB	0.523	0.16	3.218	0.20	0.84	3321	0.001 **
BR*TWTA	0.033	0.009	3.52	0.014	0.501	3320	0.0004 ***
BR*TWTB	0.024	0.009	2.54	0.005	0.041	3320	0.01 *
Mean Hb (μM)							
Intercept	0.65	0.46	1.469	−0.23	1.53	42.69	0.149
Age	0.00001	0.003	0.004	−0.006	0.006	38.59	0.99
BR	−0.0018	0.01	−1.044	−0.052	0.016	43.88	0.58
Vo2 max	−0.12	0.23	−0.553	−0.557	0.316	39.85	0.30
Task: TWTA	−0.272	0.189	−1.442	−0.64	0.097	3323	0.149
Task: TWTB	−0.679	0.189	−3.598	−1.05	−0.31	3323	0.0003 ***
BR*TWTA	−0.021	0.01	−1.956	−0.04	0.0004	3322	0.05
BR*TWTB	−0.005	0.01	−0.487	−0.026	0.015	3322	0.62

Note: Significance set at * *p* < 0.05, ** *p* < 0.01, *** *p* < 0.001. Abbreviations: SE = standard error; df = degree of freedom, CI = confidence intervals. HbO_2_ = oxygenated hemoglobin; Hb: deoxyhemoglobin; TWT_A_ and TWT_B_: Trail Walking Tasks A and B; Vo2 max: fitness levels.

## Data Availability

Research data will be available upon request by authors.

## Abbreviations

The following abbreviations are used in this manuscript:

fNIRS	Functional Near-Infrared Spectroscopy
TWT	Trail Walking Task
HbO_2_	Oxygenated Hemoglobin
Hb	Deoxygenated Hemoglobin
PFC	Prefrontal Cortical Activation
MoCA	Montreal Cognitive Assessment
Mini-BEST	Mini-Balance Evaluations Systems Test
BR	Breathing Rate
